# A Useless Dispute Settled

**Published:** 1866-11

**Authors:** 


### A USELESS DISPUTE SETTLED.

THE question of veracity, or "apprehension," between the "J. H. McQ." editor of the "Cosmos" and myself, came to a happy termination at the Boston meeting, by his cordially informing me that the official phonographic report confirmed my statements. I hoped he would state the same in the "Cosmos;" but as several numbers have appeared since, I suppose he has forgotten it. The readers of the "Register" have a right to know that I do not state for *fact* that which is only supposition, and hence this notice. W.

